# Draft Genomic Sequences of Pseudomonas moorei Strains Isolated from Wild Cranberry Bogs in Massachusetts

**DOI:** 10.1128/mra.00650-22

**Published:** 2022-09-22

**Authors:** Nima Sadeghi, Kiranpreet Kaur, Scott Soby

**Affiliations:** a Arizona College of Osteopathic Medicine, Midwestern University, Glendale, Arizona, USA; b Biomedical Sciences, College of Graduate Studies, College of Veterinary Medicine, Midwestern University, Glendale, Arizona, USA; SIPBS, University of Strathclyde

## Abstract

Pseudomonas moorei has been used to detoxify recalcitrant environmental contaminants from the pharmaceutical industry. Two *P. moorei* strains were isolated from soil in the pristine wild cranberry bogs of the Cape Cod National Seashore that putatively encode genes for degradation of 4- and 5-*chloro*salicylates, acetaminophen, and diclofenac.

## ANNOUNCEMENT

Pseudomonas moorei, first isolated from Elbe river sediments in Germany, is notable for its ability to detoxify pharmaceutical industry environmental contaminants such as 4- and 5-chlorosalicylates, acetaminophen, and diclofenac (1–3), but nothing is known about the activities of these bacteria in pristine ecosystems. Two *P. moorei* strains were isolated from dune swale bog soils as part of a culture-dependent survey of bacteria within the Cape Cod National Seashore (42.070624 N, 70.210548 W and 42.064742 N, 70.117562 W) in early July 2012. Samples that were ≈2g from 5 cm-deep soil cores were vortexed in sterile water, the rinsate was plated on King’s medium B (KMB) supplemented with 50 μg mL^−1^ ampicillin and cycloheximide, and incubated for 48 h at 26°C. Long-wave UV fluorescent colonies were colony-purified 3× on KMB, and stored in 34% glycerol at −80°C. Kits were used as per manufacturer instructions. Populations of MWU12-2021 and MWU12-2302 were inoculated into overnight KMB broth cultures and gDNA was extracted with a DNeasy blood and tissue kit (Qiagen, USA). Illumina-compatible libraries were constructed with a Hyperplus library preparation kit (Kapa Biosystems KK8514, Roche, USA). DNA was enzymatically sheared to ≈500 bp fragments, end-repaired, then A-tailed. Illumina-compatible adapters with unique indexes (IDT 00989130v2, Coralville, Iowa) were ligated to each sample, cleaned using Kapa Biosciences pure beads (KK8002), and amplified with HiFi enzyme (Kapa, KK2502). Library fragment sizes were determined with an Agilent Tapestation and quantified by qPCR with a Kapa library quantification kit KK4835; QuantStudio 5 (Thermo Fisher Scientific, USA). Libraries were then multiplex-pooled and sequenced on a 2 × 250-bp flow cell in an Illumina MiSeq. Default parameters were used for all software except as noted. Raw reads were assembled with Unicycler v0.4.8 (4) and polished with Pilon v1.23 (5) within the PATRIC Comprehensive Genome Analysis pipeline v3.6.12 (http://patricbrc.org) using default settings except for the trim setting, which was set to “true” (6). Trim Galore v0.4.0 and QUAST v5.0.2 (7, 8) were used for adapter trimming and quality control. Genome sequences were annotated using RASTtk v1.073 (9) as part of the PATRIC pipeline. Isolates MWU12-2021 and MWU12-2302 were determined with high confidence as *P. moorei* by GBDP phylogeny with the Type (Strain) Genome Server v342 (https://tygs.dsmz.de/) (Table 1) (10). Both isolates contain genes that are predicted to encode dienelactone hydrolases as key enzymes in *chloro*salicylate degradation, as well as catechol 1,2-dioxygenases and homogentisate 1,2-dioxygenases, which are involved with acetaminophen and diclofenac catabolism. Although the two isolates are similar, only MWU12-2021 is predicted to contain ring-hydroxylating dioxygenases. Like the type isolate *P. moorei* RW10^T^, the wild cranberry bog isolates thus contain putative genes for enzymes that would enable them to degrade 4- and 5-*chloro*salicylates, acetaminophen, and diclofenac.

**TABLE 1:** Data summary

Isolate	BioSample	GenBank accession	SRA accession	Genome size (bp)	No. of contigs	N_50_ (bp)	G+C content (%)	Total read length (bp)	No. of reads (x10^6^)	Read length (bp)	Coverage (x)	Coding sequences	dDDH^*a*^
MWU12-2021	SAMN27566574	JALPRM000000000	SRR18940314	6,454,086	94	327,371	59.59	771,173,334	3.51	219	119	6,061	85.0
MWU12-2302	SAMN27187300	JALJSI000000000	SRR18576229	6,699,168	91	221,423	59.36	866,302,177	3.67	233	129	6,308	84.6

a dDDH_d4_ values were calculated by comparison with *P. moorei* type strain RW10^T^ (DSM12647; FNKJ00000000).

### Data availability.

The whole-genome shotgun project has been deposited in DDBJ/EMBL/GenBank BioProject PRJNA691338 under the genome and SRA accession numbers in Table 1. The versions described in this paper are JALPRM000000000.1 and JALJSI000000000.1. RASTtk annotations are available under open license at Zenodo.com (https://zenodo.org/record/6459110#.YrZTmbfMKUk and https://zenodo.org/record/6518887#.YrZUGbfMKUk).
